# Internal Hemorrhoids: A Source of Massive Obscure Lower Gastrointestinal Bleeding in Cirrhosis

**DOI:** 10.7759/cureus.28138

**Published:** 2022-08-18

**Authors:** Benjamin G Morrison, Trevor C Morris, Caleb W Phillips, Hirotaka Kato

**Affiliations:** 1 College of Medicine, University of Kentucky, Lexington, USA; 2 Internal Medicine, University of Kentucky, Lexington, USA

**Keywords:** hematochezia, lower gastrointestinal hemorrhage, obscure gi bleeding, rectal bleeding, internal hemorrhoids

## Abstract

Anorectal bleeding is the second most common site of lower gastrointestinal bleeding. Colonoscopy remains the gold standard test to localize sources of lower gastrointestinal bleeding, but it can miss left-sided colon pathologies such as diverticula, rectal varices, and internal hemorrhoids. We report an unusual case of a male cirrhotic patient with massive hemorrhoidal bleeding which went undiagnosed despite multiple imaging and endoscopic evaluations. He underwent urgent sigmoidoscopy that identified grade III internal hemorrhoids and sclerotherapy which resolved the hematochezia. Decompensated cirrhosis complicates patient candidacy for surgical hemorrhoidectomy, but sclerotherapy is a viable option even for high-risk patients. Urgent sigmoidoscopy during active bleeding should be considered if hemorrhoidal bleeding is suspected but inconclusive by colonoscopy.

## Introduction

Anorectal bleeding is the second most common site of lower gastrointestinal (GI) bleeding following diverticular disease [1]. Hemorrhoidal bleeds, specifically, are diagnosed in up to 21% of patients admitted to hospitals with a presentation of lower GI bleeding and are usually mild and self-limiting [1]. Massive bleeds from hemorrhoids do occur but are mostly described in elderly patients on anticoagulation [2]. Traditionally, colonoscopy is regarded as the gold standard in localizing the source of lower GI bleeds, especially from hemorrhoidal sources, but may have reduced sensitivity (70%) during active extravasation [3]. It is rare to have internal hemorrhoids undetectable by traditional diagnostic modalities produce massive, transfusion-dependent GI bleeding [4]. Here we report an unusual case of a cirrhotic patient with a massive internal hemorrhoidal bleed, requiring multiple blood transfusions, that avoided detection despite an extensive workup and endoscopic evaluations.

## Case presentation

A 52-year-old male with a past medical history of alcoholic cirrhosis and coronary artery disease on aspirin presented with a chief complaint of large volume bright red blood per rectum (BRBPR). The bleeding started approximately seven months prior to presentation and the patient underwent a polypectomy via colonoscopy as part of the initial workup. The procedure was complicated by persistent bleeding. Further workup established the diagnosis of alcoholic cirrhosis and BRBPR continued to occur intermittently over the next few months. The bleeding acutely worsened four weeks prior to presentation, and the patient was hospitalized at an outside hospital where he was found to have grade I esophageal varices on esophagogastroduodenoscopy (EGD) and non-bleeding internal hemorrhoids on perianal examination. Colonoscopy revealed multiple polyps without stigmata of bleeding. Computed tomography (CT) of abdomen and pelvis with intravenous contrast and nuclear RBC tagged scan found no bleeding source. The patient required four blood transfusions during that hospitalization, and ultimately no source of bleeding could be identified.

**Figure 1:**
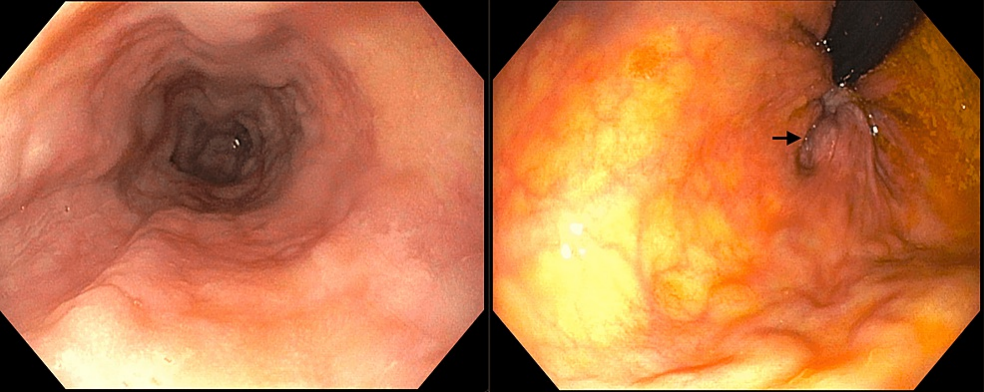
Grade I esophageal varices (left) and internal hemorrhoids (right) found by EGD and colonoscopy The arrow points to internal hemorrhoids. EGD: esophagogastroduodenoscopy

Three days following discharge, the patient was sent to our hospital for persistent BRBPR by his primary care physician. On presentation, he was afebrile at 36.4 degrees Celsius, blood pressure normotensive at 129/67 mmHg, and tachycardic at 106 beats per minute. Initial laboratory tests were remarkable for hemoglobin 8.3 g/dL (reference range 13.7-17.5), platelet 174 x 10^3^/uL (155-369), international normalized ratio 1.6 (0.9-1.1), and bilirubin 22.8 mg/dL (0.2-1.1) (see Table 1). Repeat EGD and colonoscopy were performed during this admission but only grade I esophageal varices, multiple non-bleeding sessile polyps, and small internal hemorrhoids without stigmata of bleeding were identified (see Figure 1). Colorectal surgery was also unable to find evidence of bleeding on rectal examination and recommended CT angiography, which was negative for an acute bleed.

**Table 1:** Initial laboratory test results WBC: white blood cell, CO2: serum bicarbonate, BUN: blood urea nitrogen, AST: aspartate transaminase, ALT: alanine transaminase, ALP: alkaline phosphatase, T-Bil: total bilirubin, TP: total protein, PT: prothrombin time, INR: international normalized ratio, aPTT: activated partial thromboplastin time.

Test	Value	Unit	Reference	Test	Value	Unit	Reference
Hematology				Chemistry			
WBC	11.3	10^3^/uL	(3.7-10.3)	AST	122	U/L	(12-40)
Hemoglobin	8.3	g/dL	(13.7-17.5)	ALT	42	U/L	(11-41)
Hematocrit	26.4	%	(40-51)	ALP	139	U/L	(40-115)
Platelet	174	10^3^/uL	(155-369)	T-Bil	22.8	mg/dL	(0.2-1.1)
Chemistry				Albumin	3.1	g/dL	(3.5-5.2)
Sodium	130	mmol/L	(136-145)	TP	6.5	g/dL	(6.3-7.9)
Potassium	4.3	mmol/L	(3.7-4.8)	Calcium	7.7	mg/dL	(8.9-10.2)
Chloride	101	mmol/L	(97-107)	Lactate	1.9	mmol/L	(0.5-2.2)
CO2	12	mmol/L	(22-29)	Coagulation			
BUN	16	mg/dL	(7-21)	PT	19	sec	(12.0-14.3)
Creatinine	1.14	mg/dL	(0.8-1.3)	INR	1.6		(0.9-1.1)
Glucose	109	mg/dL	(74-99)	aPTT	39	sec	(25-35)

**Figure 2:**
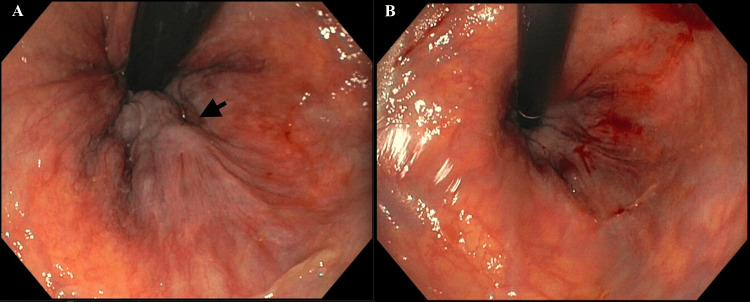
Grade III internal hemorrhoids before (A) and after (B) sclerotherapy The arrow points to grade III non-bleeding internal hemorrhoids.

However, the patient’s BRBPR continued to persist and required intermittent transfusions. His bleeding was sporadic and only with defecation, raising suspicion that his symptoms were due to hemorrhoids, even though prior scopes visualized small hemorrhoids only. The patient underwent further investigation with urgent flexible sigmoidoscopy and large, protruding, non-bleeding grade III internal hemorrhoids were discovered and subsequently treated with sclerotherapy, resolving his bleeding (see Figure 2). Hemoglobin remained stable for the remainder of his admission, and he was discharged in the following days.

## Discussion

Hemorrhoids affect nearly 4.4% of the United States population each year [5]. Internal hemorrhoids specifically present as bright red blood at the end of defecation secondary to straining; while usually associated with mild bleeding, they have been known to cause large volume blood loss, particularly in the setting of anticoagulant and antiplatelet use [2,6]. It is critical for clinicians to be aware that internal hemorrhoidal bleeding can occur intermittently with defecation [5]. Utilizing colonoscopy to identify lower GI bleeding is still considered the gold standard for detection, but massive internal hemorrhoidal bleeding can remain undiagnosed by colonoscopy. Colonoscopy is known to miss left-sided pathologies such as diverticula [7] and rectal varices [8]. In our case, colonoscopies continued to visualize only "small" hemorrhoids but the severity of his internal hemorrhoids went undiscovered until flexible sigmoidoscopy could be completed. CTs and nuclear RBC tagged scan were unremarkable likely because the bleeding occurred intermittently with defecation. It is important to remember that colonoscopy can miss or misdiagnose the severity of internal hemorrhoids as was in our case.

Ibrahim et al. reported five cases of obscure GI bleeding. In three out of these five cases, unprepared sigmoidoscopy right after the bleeding episode identified internal hemorrhoids as the source of bleeding [4]. All five cases underwent surgical hemorrhoidectomy. A commonality was that these cases had multiple endoscopies and/or scans prior, characteristics of anorectal bleed (e.g., “blood squirting from the anal canal”) and a history of chronic obscure GI bleeding longer than one year. None of these bleeding cases had cirrhosis as a complicating factor, thus our case provides a unique contribution to literature as internal hemorrhoids should be considered as massive, recurrent, and transfusion-dependent bleeding in cirrhotic patients without rectal varices.

For detection and treatment, sigmoidoscopy provides the benefit of not only visualization of internal hemorrhoids but simultaneously allowing intervention with sclerotherapy. Since sclerotherapy has become a reliable treatment option for internal hemorrhoids with advances in endoscopic interventions [9], patients do not necessarily need to go through surgery or proctoscopy under anesthesia following diagnostic endoscopic examination. Moreover, patients with decompensated cirrhosis are high-risk surgical candidates, so access to newer endoscopic interventions is quite beneficial. Even though surgical treatment such as stapled hemorrhoidopexy has been reported as a safe option in patients with Child-Pugh class A or B cirrhosis [10], it would be best to avoid surgery in more advanced cirrhosis like ours.

## Conclusions

Internal hemorrhoids are a common cause of rectal bleeding, that can be missed or misclassified on routine colonoscopy. Urgent sigmoidoscopy during active bleeding should be considered if hemorrhoidal bleeding is suspected but inconclusive. Even if there is no evidence of active bleed on visualization, internal hemorrhoids should still be considered as the source of bleeding in cases of large volume BRBPR or symptoms suggestive of anorectal bleeding.
